# Androgen Receptor and Its Splicing Variant 7 Expression in Peripheral Blood Mononuclear Cells and in Circulating Tumor Cells in Metastatic Castration-Resistant Prostate Cancer

**DOI:** 10.3390/cells9010203

**Published:** 2020-01-14

**Authors:** Mercedes Marín-Aguilera, Natalia Jiménez, Òscar Reig, Ruth Montalbo, Ajit K. Verma, Giancarlo Castellano, Lourdes Mengual, Iván Victoria, María V. Pereira, Maria Milà-Guasch, Susana García-Recio, Daniel Benítez-Ribas, Raquel Cabezón, Azucena González, Manel Juan, Aleix Prat, Begoña Mellado

**Affiliations:** 1 Translational Genomics Group and Targeted Therapeutics in Solid Tumors, Institut d’Investigacions Biomèdiques August Pi i Sunyer (IDIBAPS), 08036 Barcelona, SpainRuthmontalbocalafell@gmail.com (R.M.);; 2 Fundació Clínic per a la Recerca Biomèdica, 08036 Barcelona, Spain; 3 Medical Oncology Department, Hospital Clínic, 08036 Barcelona, Spain; 4 Department of Human Oncology, University of Wisconsin-Madison, Madison, WI 53706, USA; 5 Genomic Unit, Institut d’Investigacions Biomèdiques August Pi i Sunyer (IDIBAPS), 08036 Barcelona, Spain; 6 Department and Laboratory of Urology, Hospital Clínic, Institut d’Investigacions Biomèdiques August Pi i Sunyer (IDIBAPS), Centre de Recerca Biomèdica CELLEX, 08036 Barcelona, Spain; 7 Department of Biomedicine, University of Barcelona, 08007 Barcelona, Spain; 8 Immunology Department, Hospital Clínic, 08036 Barcelona, Spain; 9 Department of Medicine, University of Barcelona, 08036 Barcelona, Spain

**Keywords:** abiraterone, androgen receptor, androgen receptor splicing variant 7, castration-resistant prostate cancer, enzalutamide, peripheral blood mononuclear cells, taxanes

## Abstract

Androgen receptor (AR) signaling remains crucial in castration-resistant prostate cancer (CRPC). Since it is also essential in immune cells, we studied whether the expression of AR full-length *(ARFL)* and its splicing variant *ARV7* in peripheral blood mononuclear cells (PBMC) predicts systemic treatment response in mCRPC in comparison with circulating-tumor cells (CTC). We measured *ARFL* and *ARV7* mRNA in PBMC and CTC from patients prior to receiving abiraterone (AA), enzalutamide (E), or taxanes by a pre-amplification plus quantitative reverse-transcription PCR. They were also tested in LNCaP-*ARV7*-transfected and in 22RV1 docetaxel-resistant (22RV1DR) cells. We studied 171 PBMC from 136 patients and from 24 non-cancer controls, and 47 CTC from 22 patients. High PBMC *ARV7* levels correlated with worse AA/E and better taxane response. In taxane-treated patients high PBMC *ARFL* also correlated with longer progression-free survival (PFS). High *ARV7* and *ARFL* expression were independently associated with better biochemical-PFS. Conversely, high CTC *ARV7* and *ARFL* correlated with shorter radiological-PFS and overall survival, respectively. High *ARV7* in 22RV1DR and LNCaP-*ARV7* cells correlated with taxane resistance. In conclusion, *ARFL* and *ARV7* at PBMC or CTC have a different predictive role in the taxane response, suggesting a potential influence of the AR pathway from PBMC in such response modulation.

## 1. Introduction

Androgen receptor (AR) signaling activation plays an important role in the progression of metastatic castration-resistant prostate cancer (CRPC). Targeting AR with the androgen synthesis inhibitor abiraterone (AA) and the AR signaling inhibitor enzalutamide (E), has shown survival benefit in such diseases [1,2,3,4]. Taxanes have also shown an inhibitory effect over AR signaling apart from their anti-microtubule effect [5].

Constitutively active AR splicing variants have been identified as an important mechanism of the AR pathway activation in conditions of androgen depletion [6]. The most studied form has been the variant 7 (*ARV7*), which lacks the AR ligand-binding domain and is constitutively activated in absence of androgens [6]. Detected in circulating tumor cells (CTC) [7] or in whole blood [8,9] *ARV7* has been associated with lower AA/E activity. However, controversial studies report its role as a biomarker of taxane response [10,11,12,13].

Alternative splicing is a normal process in vertebrates and it is correlated with the complexity of the organism [14,15]. AR splicing variants have been identified in healthy human tissues and it has been speculated that the conservation of the AR splicing pattern in different tissues and in evolutionarily distant vertebrate species could indicate the functional importance of these AR forms [14].

Because AR is expressed in blood cells, this tissue has been demonstrated to be feasible for diagnosing genetic disorders affecting AR, such as androgen insensitivity syndrome [16]. Peripheral blood mononuclear cells (PBMC) mainly consist of lymphocytes and monocytes, but may also contain CTC. In prior work, we showed that the expression of deregulated prostate cancer genes can be detected in PBMC from patients with mCRPC [17]. Specifically, the detection of specific prostate cancer genes such as *TMPRSS2-ERG* could act as a potential biomarker of taxane resistance [18,19].

In this study we show the non-prostate cancer-specific detection of *ARV7* mRNA in PBMC. Moreover, our results suggest a different role of *ARV7* mRNA in taxane response when detected in PBMC vs. CTC samples in mCRPC patients.

## 2. Materials and Methods

### 2.1. Design and Sensitivity Measurement of ARV7 Detection

**Figure 1:**
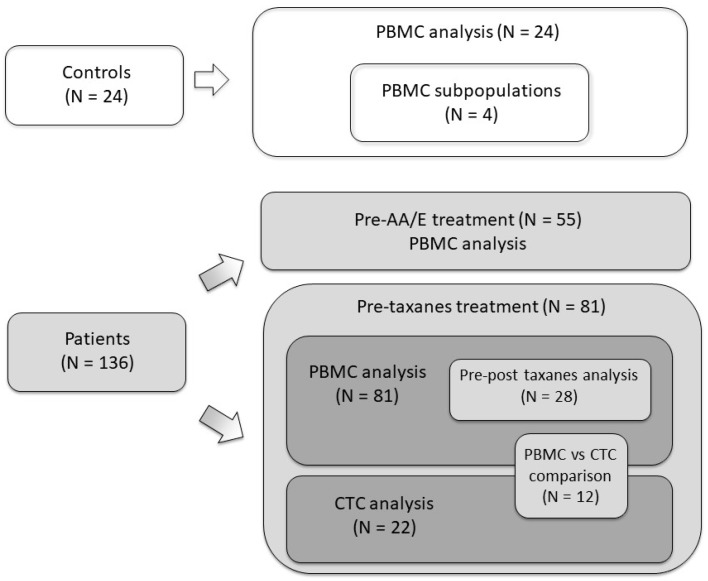
Scheme of patients included in this study. PBMC: peripheral blood mononuclear cells; AA/E: airaterone/enzalutamide; CTC: circulating tumor cells; N: number of patients.

Primer Express software v3.0 was used to design a primer/probe set to detect *ARV7*. Specifically, TaqMan probe was designed for the exon 3-cryptic exon 3 (CE3) boundary. The sequence of the selected probe was 5′-tgggagaaaaattccgg-3′. For selected primers the forward sequences was 5’-gaaatgttatgaagcagggatgact-3’ and the reverse sequence was 5’-ggtcattttgagatgcttgcaa-3’. Using this pair of primers, we expected an amplicon of 73 bp length. The sensitivity of the assay was tested by diluting 22RV1 cells (which express *ARV7*) from 0 to 2000 cells in 1 × 10^6^ PC-3 cells (which do not express *ARV7*) (Figure 1). RNA extraction and quantitative reverse-transcription PCR (qRT-PCR) were performed as described below. To confirm the *ARV7* sequence, amplicons obtained by qRT-PCR from 22RV1 cell line (positive control), PBMC from three controls, and three CRPC patients were cloned and sequenced. Specifically, the 73 bp PCR products were purified with PureLink Quick Gel Extraction Kit (Invitrogen, Waltham, MA, USA) following manufacturer instructions. DNA fragments were ligated into pJET1.2/blunt vector using the sticky-end protocol from CloneJET PCR Cloning Kit (Thermo Scientific, Waltham, MA, USA). The constructs were transformed into *Escherichia coli* DH5α competent cells by heat shock and plated on Luria-Bertani agar supplemented with carbenicillin (100 µg/mL). Plasmids from single colony transformants were purified by Zyppy Plasmid Miniprep Kit (Zymo Research, Irvine, CA, USA) according to manufacturer recommendations. The *ARV7* amplicon was finally confirmed by sequencing the plasmids with pJET1.2 forward and reverse primers (Beckman Coulter Genomics, Indianapolis, IN, USA).

### 2.2. Patients, Controls, and Samples

Men with mCRPC, according to Prostate Cancer Working Group 2 (PCWG2) criteria [20], who were candidates for AA, E, or taxanes were eligible for the present study. Twenty-four non-cancer individuals (20 men and four women; mean age 45.2 years (range 23.8–72.4 years) were included as negative controls; nine were healthy volunteers and 15 were admitted at the hospital for non-oncologic surgery (seven urinary lithiasis, three urinary incontinence, two renal transplantation, two penile prosthesis, and one urethral stenosis). Four of them were used for PBMC subpopulation analysis (Figure 1). Patients were treated with E 160 mg/day orally, AA 1000 mg/day orally, or docetaxel 75 mg/m^2^ intravenous every 3 weeks, the last two in association with prednisone 10 mg/day orally until unacceptable toxicity or progression. Disease progression and treatment response were defined according to PCWG2 criteria [20,21]. PSA levels were measured monthly. Computed tomography and bone scans were performed every two to four months or when clinically indicated. PSA-progression-free survival (PSA-PFS), radiologic-PFS (RX-PFS), and overall survival (OS) were calculated from the date of treatment initiation to PSA progression, RX progression, and death or last follow-up visit, respectively. The study was conducted in accordance with the Declaration of Helsinki, and the protocol was approved by the Ethics Committee of Hospital Clinic (Code HCB/2015/0342). All participants provided written informed consent.

### 2.3. PBMC Subpopulation Isolation and TCD4+ Selection

Five peripheral blood samples (10 mL/each) from four non-oncologic controls were collected in EDTA–containing vacutainers (Sarstedt, Nümbrecht, Germany). Magnetic isolation through negative selection of CD4 and CD8 T-cells, B-lymphocytes, monocytes, and T-natural killer cells (NK) was performed using the automated MACS technology (Miltenyi Biotec, Bergisch Gladbach, Germany). Furthermore, CD4 T-cells from four blood donors were isolated by using the human TCD4+ cell isolation kit (Miltenyi Biotec), following manufacturer’s instructions. T-cells were stimulated with α-CD3/CD28 beads (Life Technologies, Carlsbad, CA, USA) for three days. After 24 h of seeding cells, they were treated with 5 nM of 5α-dihydrotestosterone (DHT; Sigma-Aldrich, St. Louis, MO, USA) and 60 uM of enzalutamide (Deltaclon, Madrid, Spain) in monotherapy and combined for 48 h by triplicate.

### 2.4. PBMC Isolation, RNA Extraction and qRT-PCR

Before treatment initiation, peripheral blood samples (10 mL) were collected in Monovette EDTA–containing vacutainers (Sarstedt). A prior tube with 5 mL of blood was extracted and discarded to avoid epithelial contamination during venipuncture. Samples were kept at 4 °C for up to 2 h until processing. Blood specimens were layered onto 10 mL of Ficoll-Paque (GE Healthcare Life Sciences, Issaquah, WA, USA). After centrifugation, PBMC were isolated, and total RNA was extracted using TRI-Reagent (Thermo Fisher Scientific, Waltham, MA, USA) according to manufacturer instructions. RNA was quantified by ND-1000 spectrophotometer (Nanodrop Technologies, Thermo Fisher Scientific). Then, 0.5 ug of total RNA was reverse transcribed using the High Capacity cDNA Archive Kit (Thermo Fisher Scientific) following manufacturer instructions. Target genes were preamplified for 14 cycles, following manufacturer instructions for the TaqMan PreAmp Master Mix Kit (Thermo Fisher Scientific), except that the final volume of the reaction was reduced to 25 μL. QRT-PCR was performed in a StepOnePlus Real-Time PCR system (Life Technologies) according to manufacturer recommendations. Data were acquired using SDS Software 1.4. Amplification reactions were performed in duplicate. Expression values were based on the quantification cycle (Cq) from target genes relative to the Cq of *GUSB* endogenous gene. Commercial codes for primers and probes were used to amplify *ARFL*, *KLK3, PTPRC,* and *GUSB* genes (Hs00907244_m1, Hs03063374_m1, Hs00236304_m1, and Hs99999908_m1, respectively; Thermo Fisher Scientific). A specific combination of primers and TaqMan probe for detecting *ARV7* was used and described above.

### 2.5. CTC Enrichment

Blood CTC enrichment was performed using the IsoFlux System (Fluxion Biosciences, South San Francisco, CA, USA). Two 10 mL EDTA tubes were collected from patients before taxane initiation and after three cycles or at progression. One of the tubes was used for CTC counting with the IsoFlux CTC Enumeration Kit, according to manufacturer’s instructions. CTC were defined as nucleated cells, morphologically intact, cytokeratin positive, and CD45 negative cells. The second EDTA tube was used for gene expression analysis. Briefly, PBMC were isolated by Ficoll (GE Healthcare, Uppsala, Sweden) gradient and incubated with anti-epithelial cell adhesion molecule (EpCAM), prostate stem cell antigen (PSCA), and N-cadherin (CDH2)-coated beads for 2 h at 4 °C. Cell–bead complexes were loaded into Isoflux cartridges to be isolated automatically by the instrument. Cells returned by the instrument were stained or total RNA was extracted and qRT-PCR with cDNA preamplification was performed as described above, using *ACTB* (Hs99999903_m1) as endogenous gene.

### 2.6. In Vitro Experiments

LNCaP cells with doxycycline-inducible expression of *ARV7* and their respective control LNCaP-vector cells were kindly provided by Dr. Nancy L. Weigel [22]. 22RV1 resistant to docetaxel (22RV1DR) and their respective control cells were kindly provided by Dr. Ajit Verma [23].

Whole-cell extracts of LNCaP induced with doxycycline were prepared and Western blot performed as described previously [22] in order to confirm the inducible ARV7 expression. Mouse anti-ARV7 1:500 (Precision antibody) and GAPDH (Ref. AM4300, Ambion, Austin, TX, USA) antibodies were used. The Odyssey fluorescence system was used to detect protein signals.

Cytotoxicity of taxanes, docetaxel, and cabazitaxel (Selleckchem, Munich, Germany), was evaluated by using the Cell Titer 96 Aqueous One Solution Cell Proliferation Assay kit (Promega, Madison, WI, USA) according to manufacturer’s instructions. Briefly, 10 thousand cells/well were seeded at 96-well plates and induced with 10 ng/mL doxycycline. Twenty-four hours later cells were treated with increasing concentrations of both docetaxel and cabazitaxel and cell viability was evaluated after 72 h of treatment.

### 2.7. Statistical Analysis

Fisher’s exact and Wilcoxon–Mann–Whitney tests were used to compare proportions and continuous variables, respectively. Optimal cut-off points for *ARV7* and *ARFL*, and PSA-PFS, RX-PFS, and OS were assessed by the maximally selected log-rank statistics method using the Maxstat package in R software [24]. Briefly, to run this test the smethod and pmethod were log-rank and Lau92, respectively, and the minimal and maximal proportions were 0.3 and 0.7, respectively, with an alpha error of 0.05. *ARV7* and *ARFL* expression levels were considered high or low according to the optimal cut-off points selected. PSA-PFS, RX-PFS, and OS were calculated using the Kaplan-Meier method and compared using the log-rank test. Univariate and multivariate analysis were performed with Cox regression. QRT-PCR experimental data were analyzed by Student *T*-test. All tests were 2-sided and *P* values < 0.05 were considered statistically significant. Statistical analysis was done with SPSS (v20) and R-Studio software (v3.1.1) [25].

## 3. Results

### 3.1. ARV7 Amplification Efficiency, Sensitivity, and Sequence Confirmation

**Figure 2:**
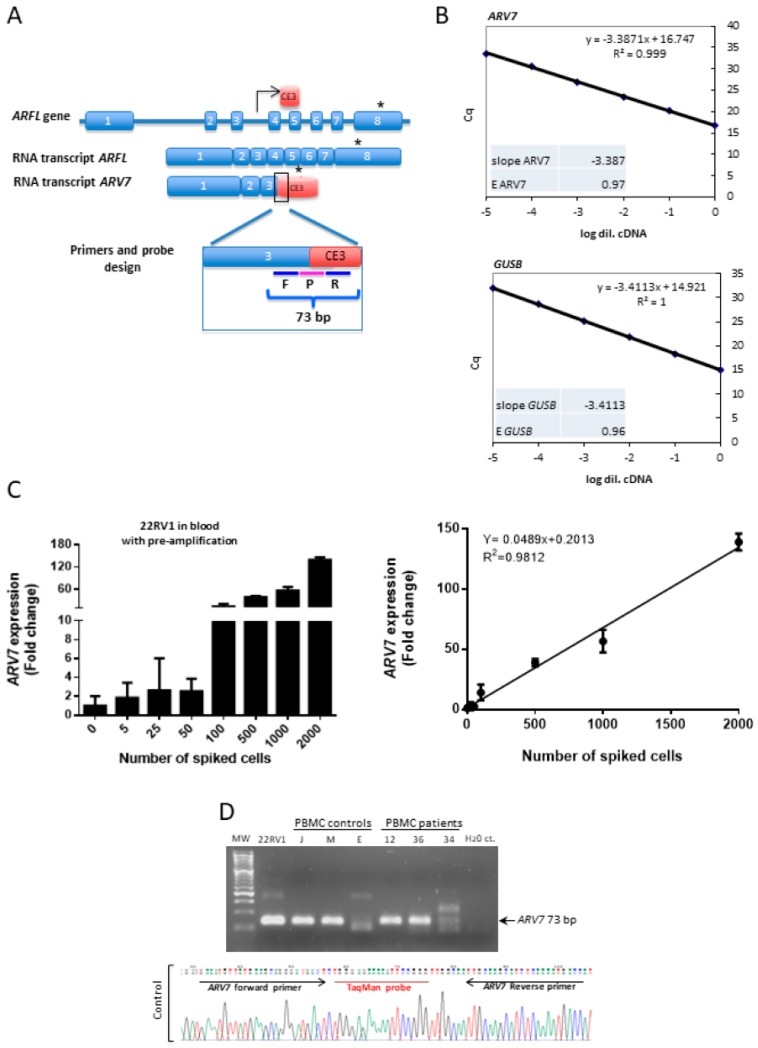
Design and sensitivity of *ARV7* detection assay. (**A**) Androgen receptor (AR) full- length (*ARFL*) gene and *ARV7* transcripts scheme that shows primers and probe design for detecting *ARV7* expression in this work. * Translation stop. F: forward primer; P: probe; R: reverse primer; CE: cryptic exon. (**B**) Standard curve with 10-fold dilution series of 22RV1 cDNA graphs showing the efficiency of the designed *ARV7* TaqMan assay in comparison with the *GUSB* assay. The efficiency ‘E’ factor was calculated according to the formula E = [10^(−1/slope)] − 1. Linear regression analysis showed the slope of the curves. Cq: quantification cycle. (**C**) Bar plot and linear regression analysis of *ARV7* detection in spiking experiments. The number of spiked cells and fold change detected are plotted on the *y*- and *x*-axis, respectively. The correlation coefficient is given at the top of the linear regression graph. (**D**) Agarose gel of *ARV7* quantitative PCR product in 22RV1 cell line (positive control) and in PBMC from three controls (J, M, and E) and three mCRPC patients (12, 36, and 34). Negative control (H_2_O ct.) was included. Sequence of one of the cloned control bands is showed. MW: 50 bp molecular marker (Niborlab).

The 22RV1 cell line, which expresses *ARV7*, was used to assess the effectiveness of the designed primers in amplification of *ARV7* (Figure 1) by cDNA pre-amplification plus qRT-PCR. Efficiency was evaluated by performing a standard curve with a 10-fold dilution series of 22RV1 cDNA. The slope of the curve for *ARV7* expression was −3.387, indicating an assay efficiency of 97.4%. *GUSB* slope and efficiency were −3.41% and 96.4%, respectively (Figure 1). Spiking experiments showed the capacity to detect *ARV7* expression if five 22RV1 cells were pooled among 1 × 10^6^ PC-3 cells (Figure 1). ARV7 amplicon was cloned into the pJET1.2/blunt vector in order to confirm its sequence in 22RV1 and in PBMC from controls and CRPC patients. The obtained sequence was the expected according to the primers used, confirming the *ARV7* detection in non-prostate cancer individuals and in patients with mCRPC (Figure 2).

### 3.2. ARV7 and ARFL Expression in PBMC from Non-Cancer Patients

To explore the basal levels of *ARV7* and *ARFL* in the non-cancer population we collected PBMC from twenty-four non-cancer individuals. *ARV7* was detected in 18 of them (75%) and *ARFL* in all the samples (100%). To further confirm the expression of *ARV7* and *ARFL* in PBMC we studied five subpopulations (CD4 and CD8 T-cells, B lymphocytes, T-natural killer cells [NK], and monocytes) isolated from PBMC of four non-cancer controls. *ARV7* mRNA was detected in T-CD4 and B-lymphocytes from two (50%) controls, in T-CD8 and NK cells from one (25%) individual, and in the monocyte subpopulation from all four (100%) controls. *ARFL* was detected in all subpopulations from all controls except for the NK population of one individual (Table 1).

**Figure 3:**
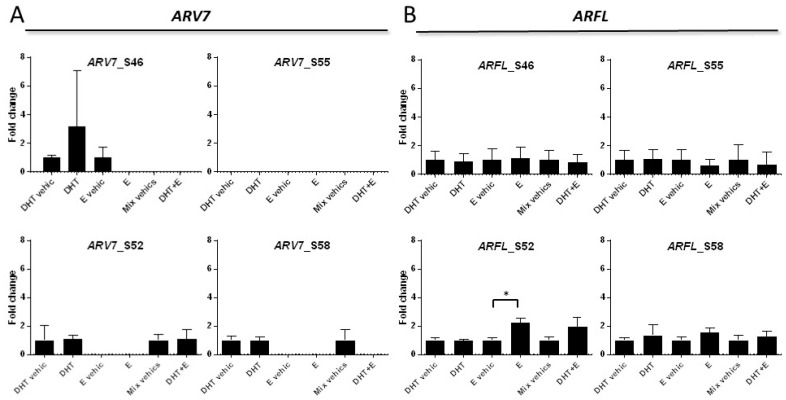
*ARV7* and *ARFL* in non-cancer CD4 T-cell subpopulation. (**A**) Bar plot representing gene expression (mean fold change +/− SD) by qRT-PCR of *ARV7* in four CD4 T-cell populations treated with 5α-dihydrotestosterone (DHT) and enzalutamide (E) individually or combined, by triplicate. Vehicles (vehic.) of treatments were methanol for DHT and DMSO for E; (**B**) Bar plot representing gene expression (mean fold change +/− SD) by qRT-PCR of *ARFL* in four CD4 T-cell populations treated with 5α-dihydrotestosterone (DHT) and enzalutamide (E) individually or combined, by triplicate. Vehicles (vehic.) of treatments were methanol for DHT and DMSO for E; *T*-test, * *P* < 0.05.

To explore the potential effect of anti-androgen treatment on *ARV7* and *ARFL* mRNA expression in PBMC we exposed a subpopulation (CD4 T-cells) from non-cancer controls to DHT or E. With the experimental conditions used, we showed that E significantly increased the expression of *ARFL* in one (25%) of the four controls (2.21-fold relative to E vehicle; *P* < 0.05) while non-significant changes were observed in *ARV7* and *ARFL* expression with DHT or E treatment (Figure 3).

### 3.3. ARV7, ARFL, KLK3, and PTPRC (CD45) Expression in CTC-Enriched Samples

**Figure 4:**
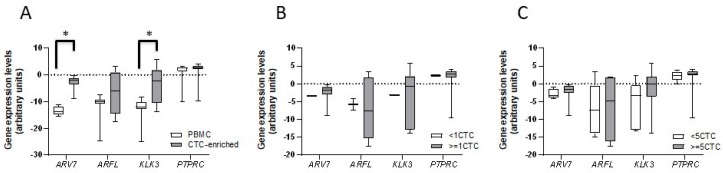
Gene expression comparison between PBMC and CTC-enriched samples. (**A**) Box plots representing gene expression (average +/− SD) by qRT-PCR of *ARV7*, *ARFL*, *KLK3,* and *PTPRC* markers in PBMC and in circulant tumoral cell (CTC)-enriched samples; (**B**) Box plots representing gene expression (average +/− SD) by qRT-PCR of *ARV7*, *ARFL*, *KLK3,* and *PTPRC* markers in CTC-enriched samples with <1 CTC vs ≥1 CTC; (**C**) Box plots representing gene expression (average +/− SD) by qRT-PCR of *ARV7*, *ARFL*, *KLK3,* and *PTPRC* markers in CTC-enriched samples with <5 CTC vs. ≥5 CTC; *T*-test, * *P* < 0.05.

In order to compare the gene expression from CTC and PBMC, in 12 patients (randomly selected) blood extraction for PBMC and for CTC-enrichment was simultaneously performed and tested for the expression of *ARV7*, *ARFL*, *KLK3,* and the white blood cell marker CD45 (PTPRC). In two patients (16.7%) no CTC were detected; the remaining 10 (83.3%) had a median CTC count of 8.5 (range 1–22). Similar expression of *PTPRC* was detected in all samples (Figure 4), confirming that the CTC-enriched samples contain contaminating white blood cells. *ARV7*, *ARFL,* and *KLK3* mRNAs were detected in all samples in both PBMC and CTC. Significantly higher levels of *ARV7* and *KLK3*, but not *ARFL*, expression were found in CTC samples, compared to PBMC (Figure 4). Although non-significant, higher levels of the prostate-specific marker *KLK3* were in CTC-positive samples (≥1 CTC vs. <1 CTC) and in samples with CTC equal to or more than five (Figure 4).

### 3.4. ARV7 and ARFL Expression in PBMC and AA/E Activity

We collected 55 PBMC samples from 55 AA/E-treated patients, 37 received AA and 18 E. Patients’ characteristics are shown in Table 2.

Median follow-up time was 14.9 months (1.5–57.9). Four (7.3%) patients primarily progressed to AA/E treatment and 29 (52.7%) responded by PSA. Median PSA-PFS and RX-PFS were 3.6 (3.2–4.1) and 6.1 (4.1–8.2) months, respectively. Median OS was 16.8 months (12.2–21.5).

**Figure 5:**
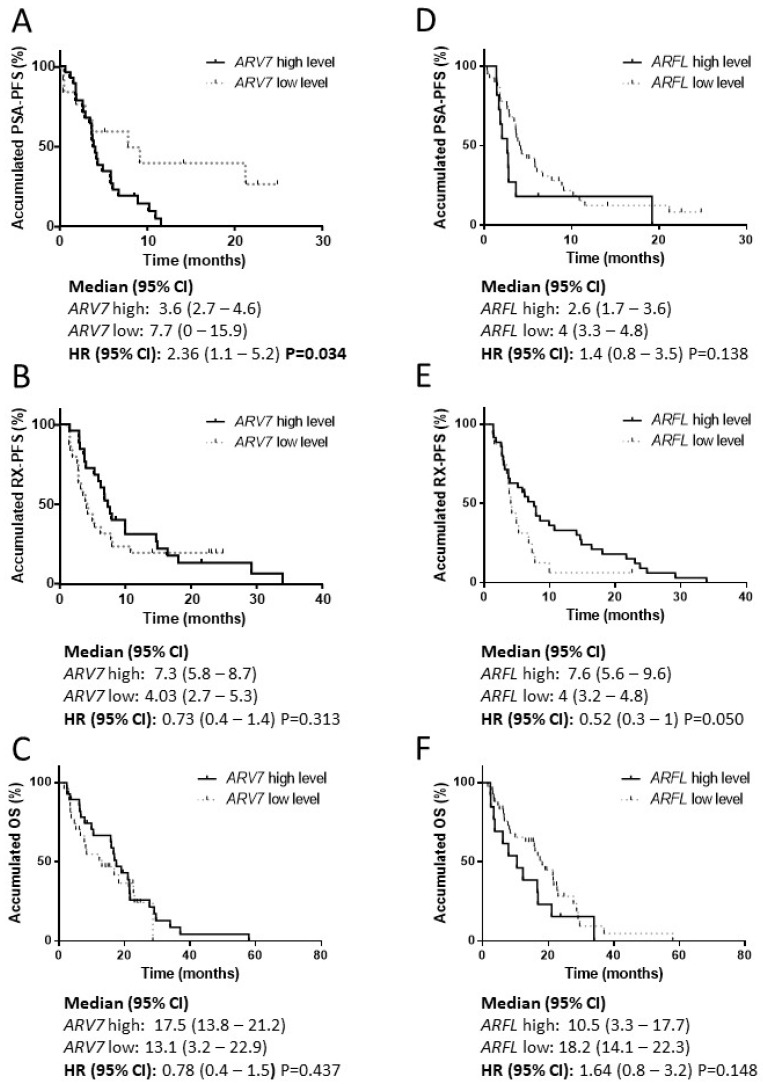
*ARFL* and *ARV7* in PBMC from AA/E-treated patients. Survival analysis in AA/E-treated patients according to *ARFL* and *ARV7* levels in PBMC samples. (**A**) Kaplan–Meier curves according to *ARV7* levels for PSA progression-free survival (PSA-PFS); (**B**) Kaplan-Meier curves according to *ARV7* levels for radiologic progression-free survival (RX-PFS); (**C**) Kaplan-Meier curves according to *ARV7* levels for overall survival (OS); (**D**) Kaplan-Meier curves according to *ARFL* levels for PSA-PFS; (**E**) Kaplan-Meier curves according to *ARFL* levels for RX-PFS; (**F**) Kaplan-Meier curves according to *ARFL* levels for OS; HR: hazard ratio; CI: confidence interval.

Samples were categorized according a selected cut-off in gene expression values, and no differences in PSA response rate were observed between patients with high or low *ARV7* (55% vs. 50%, *P* = ns) and *ARFL* expression (45.5 vs. 55.8 *P* = ns). However, high *ARV7* levels were associated with a shorter PSA-PFS (hazard ratio (HR) 2.4; 95% confidence interval (CI) 1.1–5.2; *P* = 0.034) (Figure 5) and high *ARV7/ARFL* ratio was an independent prognostic factor for adverse PSA-PFS (HR 8.5; 95% CI 1.8–39.6; *P* = 0.006) (Table 3).

### 3.5. ARV7 and ARFL Expression in PBMC and Taxane Activity

We collected 92 PBMC samples from 81 patients receiving taxanes (71 docetaxel and 10 cabazitaxel, and 11 were analyzed for both taxane treatments). Patients’ characteristics are shown in Table 2.

Median follow-up time was 13.82 months (1.37–82.27), median PSA-PFS and RX-PFS were 4.73 (3.6–5.8) and 6.43 (5.2–7.6) months, respectively. Median OS was 15.13 months (10.6–19.7). PSA response rate to taxanes was 44.6%.

**Figure 6:**
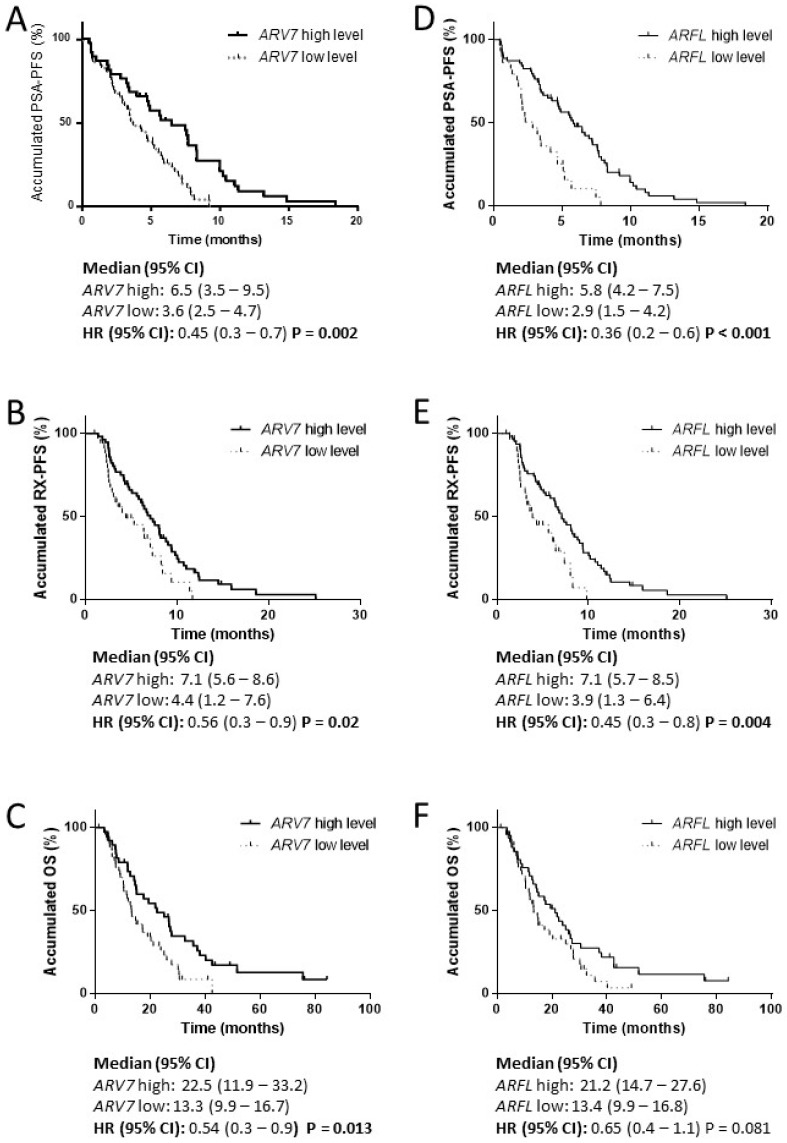
*ARFL* and *ARV7* in PBMC from taxane-treated patients. Survival analysis in taxane-treated patients according to *ARFL* and *ARV7* levels in PBMC samples pre-treatment. (**A**) Kaplan-Meier curves according to *ARV7* levels for PSA progression-free survival (PSA-PFS); (**B**) Kaplan-Meier curves according to *ARV7* levels for radiologic progression-free survival (RX-PFS); (**C**) Kaplan-Meier curves according to *ARV7* levels for overall survival (OS); (**D**) Kaplan-Meier curves according to *ARFL* levels for PSA-PFS; (**E**) Kaplan-Meier curves according to *ARFL* levels for RX-PFS; (**F**) Kaplan-Meier curves according to *ARFL* levels for OS; HR: hazard ratio; CI: confidence interval.

High *ARV7* levels correlated with a better PSA-PFS (HR 0.45, 95% CI 0.3–0.7, *P =* 0.002), RX-PFS (HR 0.56, 95% CI 0.3–0.9, *P =* 0.02) and OS (HR 0.54, 95% CI 0.3–0.9, *P =* 0.013) (Figure 6). Moreover, higher *ARFL* was associated with better outcomes for PSA-PFS (HR 0.36, 95%CI 0.22–0.61, *P* < 0.001) and RX-PFS (HR 0.45, 95%CI 0.26–0.77, *P =* 0.004), but not for OS (HR 0.65, 95%CI 0.41–1.1, *P =* 0.08) (Figure 6).

In the multivariate analysis, *ARV7* and *ARFL* were independent prognostic factors for favorable PSA-PFS (HR 0.5, 95%CI 0.3–0.9, *P* = 0.019 and HR 0.4, 95%CI 0.2–0.7, *P* = 0.001, respectively) (Table 4).

We compared baseline *ARV7* and *ARFL* expression in PBMC between both AA/E and taxane cohorts (Table 5). We observed that the % of *ARV7*-high expression samples was significantly higher in the AA/E than in the taxane cohort (74.5% vs. 41%, odds ratio 4.46, *P* < 0.001). Regarding to *ARFL* expression, taxane-cohort presented higher % of *ARFL*-high samples than AA/E-cohort patients (68.5% vs. 21.8%, odds ratio 7.78, *P* < 0.001). Percentages of high *ARV7* and *ARFL* expression according prior AA/E or taxane therapy in both cohorts are shown in Table 5.

### 3.6. Variations in ARV7 and ARFL Expression Levels in PBMC after Taxanes

**Figure 7:**
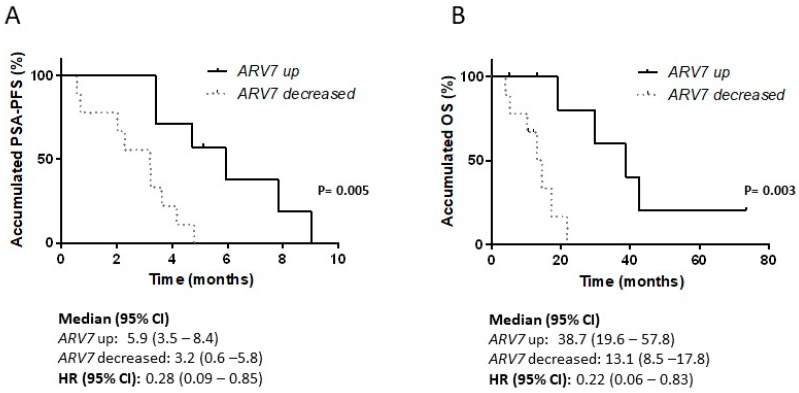
Variations in *ARV7* and *ARFL* expression levels after taxanes. Survival analysis in taxane-treated patients according to changes in *ARFL* and *ARV7* levels in PBMC samples post-taxane treatment regarding to pre-treatment. (**A**) Kaplan-Meier curves for PSA progression-free survival (PSA-PFS); (**B**) Kaplan-Meier curves for overall survival (OS); HR: hazard ratio; CI: confidence interval.

We collected 56 paired PBMC samples (pre and post taxane treatment) from 28 patients. Patients’ characteristics are shown in Table 2. We classified samples according their changes in *ARV7* and *ARFL* expression levels at post respect to pre-treatment status. Those patients whose *ARV7* mRNA levels increased higher than 1 arbitrary unit had longer PSA-PFS (5.9 vs. 3.2 months; *P* = 0.005) and OS (38.7 vs. 13.8 months; *P* = 0.003) than those whose levels decreased more than 1 unit (Figure 7). No significant results were obtained regarding to changes in *ARFL* expression in PBMC (data not shown).

### 3.7. ARV7 and ARFL Expression in CTC and Benefit to Taxanes

We also collected 24 CTC-enriched samples from 22 patients prior to receiving taxanes (16 docetaxel, 8 cabazitaxel, and two were analyzed for both taxanes treatments). Patients’ characteristics are shown in Table 1.

**Figure 8:**
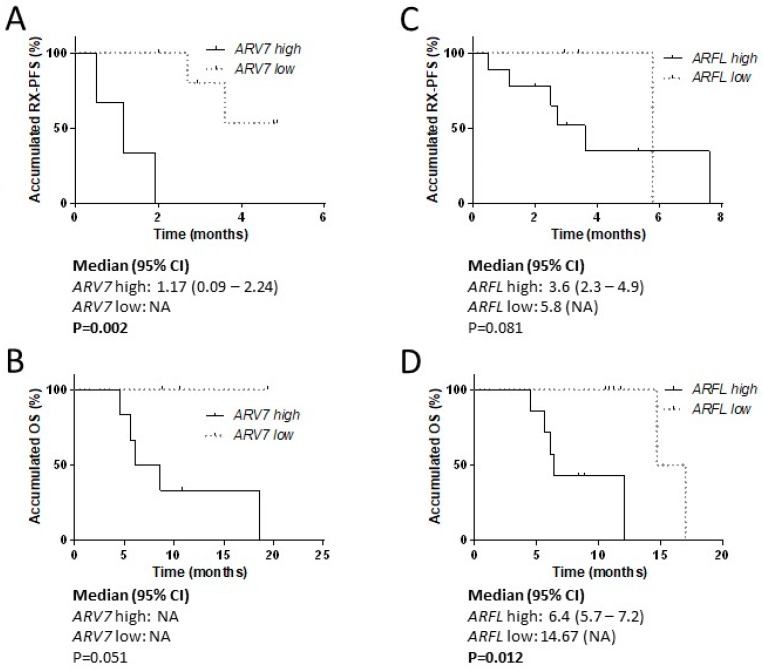
*ARFL* and *ARV7* in CTC from taxane-treated patients. Survival analysis in taxane-treated patients according to *ARFL* and *ARV7* levels in CTC samples pre-treatment. (**A**) Kaplan-Meier curves according to *ARV7* levels for radiologic progression-free survival (RX-PFS); (**B**) Kaplan-Meier curves according to *ARV7* levels for overall survival (OS); (**C**) Kaplan-Meier curves according to *ARFL* levels for RX-PFS; (**D**) Kaplan-Meier curves according to *ARFL* levels for OS. HR: hazard ratio; CI: confidence interval; NA: not achieved.

Among the 24 samples, we detected *ARV7* and *ARFL* expression in nine (37.5%) and 13 (54.2%) samples, respectively. Six of the nine (66.7%) and 10 of the 13 (76.9%) samples expressed high levels of *ARV7* and *ARFL*, respectively, according to the Maxstat cut-off. No significant differences were observed in taxane response rates between patients with high *ARV7* (0% vs. 16.7%) and *ARFL* (33.3% vs. 33.3%) levels, probably because of the small size of the cohort. However, it is of note that none of the patients with high *ARV7* expression presented PSA response. Regarding survival analysis, high *ARV7* expression correlated with worse RX-PFS (*P* = 0.002) and high *ARFL* correlated with worse OS (*P* = 0.012) (Figure 8).

### 3.8. ARV7 and Taxane Activity In Vitro

**Figure 9:**
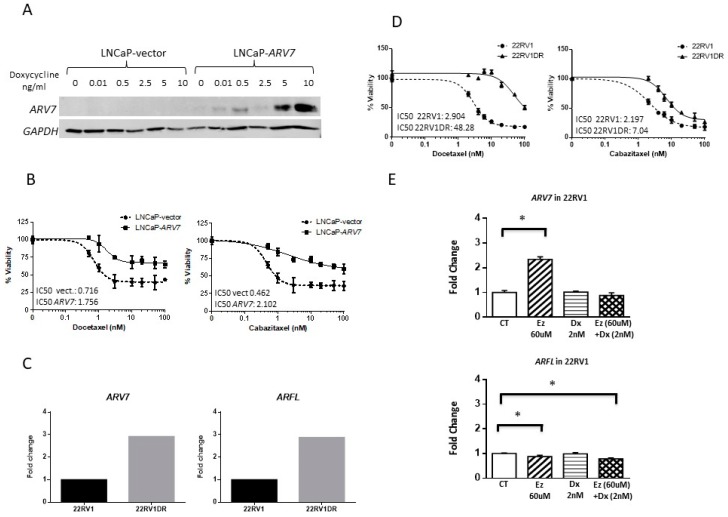
*ARV7*-induced sensitivity to taxanes in in vitro experiments. (**A**) Western blot of ARV7 expression in LNCaP-*ARV7* cells induced with different concentrations of doxycycline (ng/mL). (**B**) Viability curves in LNCaP-*ARV7* vs. LNCaP-vector cells with both docetaxel and cabazitaxel drugs in the presence of doxycycline after 72 h exposure. IC50 doses are showed. (**C**) Bar plots representing *ARV7* and *ARFL* mRNA levels in 22RV1DR cells and their parental cells measured by qRT-PCR. (**D**) Viability curves in 22RV1DR vs. 22RV1 cells with both docetaxel and cabazitaxel drugs after 72 h exposure. IC50 doses are showed. (**E**) Bar plots representing *ARV7* and *ARFL* mRNA levels in 22RV1 cells treated with enzalutamide (Ez) 60 uM and docetaxel (Dx) 2 nM, separately and in combination. CT: control. *T*-test, * *P* < 0.05.

In order to explore the role of *ARV7* specifically from tumor cells in taxane response, we performed in vitro experiments using LNCaP cells harboring doxycycline-inducible *ARV7* expression (LNCaP-*ARV7*) and 22RV1 docetaxel-resistant cells. After confirming the induction of *ARV7* expression by doxycycline treatment in LNCaP cells (Figure 9), we observed that LNCaP-*ARV7* cells were more resistant to taxanes than control cells with IC50 around 2.5- and 4.5-fold for docetaxel and cabazitaxel, respectively (Figure 9). 22RV1DR cells presented higher levels of *ARV7* and *ARFL* expression than parental cells, and were also more resistant to taxanes with IC50 around 16- and 3-fold for docetaxel and cabazitaxel, respectively (Figure 9). Parental 22RV1 cells treated with androgen deprivation therapy (enzalutamide) increased the expression of *ARV7* that was reverted by the addition of docetaxel (Figure 9). All together these data support the conclusion that *ARV7* in tumor is modulated by systemic treatment and may be associated with taxane resistance.

## 4. Discussion

In this paper we report that *ARV7* mRNA is not specific to prostate cancer but can also be found in other types of cells that normally express *ARFL,* such as monocytes or lymphocytes. We found that *ARV7* expression in PBMC predicted lower AA/E benefit but a better outcome to taxanes. Moreover, we observed a different role for the expression of *ARV7* when it was detected in PBMC or in CTC with respect to prediction of taxane outcomes suggesting that AR signaling in blood cells may influence systemic treatment response in patients with mCRPC.

It is well known that in addition to prostate tissue, *ARFL* receptor is detected in other cells in the body [26]. Such cells are also susceptible to having the *ARV7* splicing form. In this study we used a very sensitive technique combining cDNA pre-amplification with real-time PCR that allowed us to detect *ARFL* expression in different PBMC subpopulations from non-cancer controls and in all patients’ samples. To a lesser extent, we also detected *ARV7* mRNA in most of PBMC subpopulations from controls and in samples from patients, confirming that the expression of *ARV7* is not specific to prostate cancer. This result agrees with the detection of *ARV7* in PBMC of almost all samples analyzed by Qu et al. [9] and with the finding by Nimir et al. of *ARV7* exosomal mRNA in one of five (20%) healthy volunteers, by using a droplet digital PCR-based approach [27]. We assume that the sensitivity between different works varies since we and Qu et al. could detect *ARFL* expression in all the samples while Nimir et al. could detect it in only three of five healthy subjects [27]. *ARV7* expression in whole blood from non-cancer individuals was also described by Todenhöfer et al. [8] and Takeuchi et al. [28]. The non-specific detection of *ARV7* in blood cells and its potential contamination of CTC-enriched samples (as observed in our comparative series) could explain, in part, some of the discordant published results according the predictive value of *ARV7* to AA/E or taxane response [7,9,10,13,29,30,31,32].

Regarding taxanes, our results in a small cohort of CTC samples showed that patients with high *ARV7* expression presented lower RX-PFS to taxanes, which is consistent with the results of the TAXYNERGY trial [13]. Accordingly, in vitro experiments showed that *ARV7*-transfected LNCaP cells are more resistant to taxanes than parental cells, and that the taxane-resistant cell line 22RV1DR presented higher levels of *ARV7* and *ARFL* expression than parental cells. Moreover, parental 22RV1 cells treated with androgen deprivation therapy (enzalutamide) increased the expression of *ARV7*, which was reverted by the addition of docetaxel. Recently, Cato et al. showed that *ARV7* binds *ARFL* and represses the transcription of tumor suppressor genes in *ARV7*-dependent CRPC models [33]. This supports the observed adverse clinical outcome of patients with high *ARV7* expression in CTC treated with both AA/E and taxanes and suggests that *ARV7* expression in tumor cells may be an adverse prognostic factor rather than a predictive factor that may help to discriminate patients for AA/E or taxane therapies.

*ARFL* and *ARV7* expression in prostatic tumor cells is modulated by systemic treatment. It has been reported that androgen deprivation therapy increases the expression of *ARFL* in tumors as well as the transcription of *ARV7*, which is closely related to *ARFL* expression [34]. Because anti-hormonal treatment in prostate cancer is a systemic therapy, one cannot disregard that this regulation has also an effect in AR-expressing cells other than prostate cancer cells. We showed in vitro that enzalutamide significantly increased the expression of *ARFL* in one (25%) of the 4 CD4 T-cells from controls while non-significant changes were observed in *ARV7* and *ARFL* expression with DHT or enzalutamide treatment. Although our results have to be considered exploratory because of the small number of subjects studied, they support the potential modulation of *ARFL* or *ARV7* expression in PBMC by systemic treatment. Whether this modulation has as a role in therapy response and in prostate cancer evolution has not been explored.

A possible explanation for the better response associated with high *ARFL* and *ARV7* levels in PBMC could be related to the activation of the immune system lead by an active AR pathway and by taxanes. AR signaling enhances proinflammatory cytokine production by macrophages [35] which could exert an anti-tumoral activity. Moreover, AR signaling activation has been also related to an increase of *RACK1* expression, a scaffold of protein kinase C (PKC) which stabilizes its conformation and promotes the activation of several pathways such as the lipopolysaccharide-induced monocytes/macrophage activation [36]. Taxanes have been also reported as stimulators of the anticancer immune response exerting an activation of macrophage, T-cell, and NK-cell function [37,38,39]. Conversely, anti-hormonal treatment may exert a down-regulation of the PKC signaling molecule RACK1 and a decrease in monocyte activation on human PBMC [36]. According to this data we hypothesize that a synergistic effect of taxane- and AR signaling-mediated immune activation could exist in those patients with higher levels of *ARV7* in PBMC. Furthermore, the different immunomodulation exerted by anti-hormonal or chemotherapy treatment could also explain the different prognostic role of *ARV7* detected in blood cells.

In conclusion, our study suggests that the AR pathway in blood cells may have an impact on the clinical outcome of patients with mCRPC. Testing larger patient populations in further studies is required to validate these results and deeply understand the mechanisms by which *ARFL* and its splicing variant *ARV7* from blood or tumor cells are involved in mCRPC evolution.

## Figures and Tables

**Table 1:** Detection of *ARV7* and *ARFL* in PBMC subpopulations from four non-cancer controls.

	Samples (*ARV7*/*ARFL*) ^1^			
Cell Type	S1	S2	S3	S4
T-CD4 lymphocytes	+/+	−/+	+/+	−/+
T-CD8 lymphocytes	+/+	−/+	−/+	−/+
B lymphocytes	−/+	+/+	−/+	+/+
NK cells	+/−	−/+	−/+	−/+
monocytes	+/+	+/+	+/+	+/+

^1^ mRNA detection is represented by +; if mRNA was not detected is showed as −; S: sample; NK: natural killer.

**Table 2:** Baseline patients’ characteristics.

	AA/E Cohort	Taxane Cohort	CTC Cohort	Pre-Post Taxane Cohort
Number of patients (samples)	55 (55)	81 (92)	22 (24)	28 (56)
Age (years)				
Median (range)	70.21 (53.3–93.3)	62.85 (32.8–79.4)	70 (41.6–87.1)	62.85 (32.8–79.4)
Stage at diagnosis, N (%)				
<IV	15 (27.3)	33 (37.7)	5 (20.8)	12 (42.9)
IV	32 (58.2)	46 (56.8)	15 (62.5)	14 (50)
No data	8 (14.5)	2 (2.5)	4 (16.7)	2 (7.1)
Gleason sum at diagnosis, N (%)				
≤7	19 (34.5)	34 (41.9)	8 (33.3)	11 (39.3)
≥8	30 (54.5)	47 (58)	16 (66.7)	17 (60.7)
No data	6 (10.9)	-	-	-
Presence of bone metastases, N (%)				
Yes	51 (92.7)	69 (85.2)	21 (87.5)	25 (89.3)
No	4 (7.3)	12 (14.8)	3 (12.5)	3 (10.7)
Presence of visceral metastases, N (%)				
Yes	10 (18.2)	20 (24.7)	7 (29.2)	8 (28.6)
No	45 (81.8)	61 (75.3)	17 (70.8)	20 (71.4)
ECOG performance status score, N (%)				
0	14 (25.5)	19 (23.5)	1 (4.2)	4 (14.3)
1 or 2	40 (72.7)	62 (76.5)	23 (95.8)	23 (82.1)
No data	1 (1.8)	-	-	1
Baseline prostate-specific antigen (ng/mL)				
Median (range)	38.5 (0.29–3282)	60.2 (1.04–1284)	27 (1.8–479.6)	27.3 (2.8–675.5)
No data (N)	-	1	-	-
Baseline hemoglobin concentration (g/L)				
Median (range)	123 (84–146)	124 (81–151)	122 (84–498)	132 (97–145)
Baseline alkaline phosphatase (U/L)				
Median (range)	177 (53–2448)	229.5 (47–4397)	142 (54–873)	175 (50–1143)
No data (N)	4	2	-	
Baseline lactate dehydrogenase				
Median (range)	412 (62–1921)	396 (163–2954)	356 (125–949)	378 (163–949)
No data (N)	3	11	-	-
Chemotherapy treatment, N (%)				
Post	19 (34.5)	-	-	-
Pre	36 (65.5)	-	-	-
Use of AA/E, N (%)				
No	-			
Pre-chemotherapy	-	22 (27.2%)	17 (70.8)	16 (57.1)
Never or Post-chemotherapy	-	59 (72.8%)	7 (29.2)	12 (42.9)

N: number of cases; ECOG: Eastern Cooperative Oncology Group; AA/E: abiraterone/enzalutamide.

**Table 3:** Univariate and multivariate Cox model for PSA-PFS in AA/E treated patients adjusted for clinically significant variables (*P* < 0.1) in univariate analysis.

	Univariate			Multivariate		
PSA-PFS	HR	95% CI	*P*-Value	HR	95% CI	*P*-Value
*ARV7* *	2.357	(1.068–5.2)	**0.034**	0.326	(0.08–1.325)	0.117
*ARFL* *	1.718	(0.841–3.511)	0.138	-	-	-
*ARV7/ARFL* *	3.8	(1.54–9.39)	**0.004**	8.492	(1.82–39.6)	**0.006**
ECOG *	1.953	(0.976–3.908)	**0.059**	1.990	(0.89–4.41)	0.090
Stage *	1.443	(0.782–2.664)	0.241	-	-	-
GLEASON *	1.228	(0.662–2.276)	0.515	-	-	-
Visceral metastases *	1.135	(0.524–2.458)	0.748	-	-	-
PSA **	1.00	(1–1.001	0.172	-	-	-
HB **	0.961	(0.938–0.985)	**0.001**	0.964	(0.938–0.992)	**0.011**
LDH **	1.001	(1–1.002)	**0.003**	1.000	(0.999–1.002)	0.438
AP **	1.003	(1.001–1.005)	**0.004**	1.001	(0.999–1.003)	0.341

* Variables considered dichotomic; ** Variables considered continuous. ECOG: Eastern Cooperative Oncology Group; PSA: prostate-specific antigen; HB: hemoglobin concentration; LDH: lactate dehydrogenase; AP: alkaline phosphatase; HR: hazard ratio; CI: confidence interval. Significant *P*-Values are shown in bold.

**Table 4:** Univariate and multivariate Cox model for PSA-PFS in taxane-treated patients adjusted for clinically significant variables (*P* < 0.1) in univariate analysis.

	Univariate			Multivariate		
PSA-PFS	HR	95% CI	*P*-Value	HR	95% CI	*P*-Value
*ARV7* *	0.449	(0.273–0.739)	**0.002**	0.487	(0.267–0.889)	**0.019**
*ARFL* *	0.364	(0.219–0.607)	**0.000**	0.366	(0.198–0.676)	**0.001**
*ARV7/ARFL* *	0.711	(0.435–1.161)	0.173	-	-	-
ECOG *	1.479	(0.862–2.539)	0.156	-	-	-
STAGE *	0.803	(0.511–1.263)	0.343	-	-	-
GLEASON *	1.256	(0.806–1.958)	0.313	-	-	-
VISCERAL METASTASES *	0.907	(0.545–1.506)	0.705	-	-	-
PSA **	1.001	(1–1.002)	**0.005**	1.001	(1–1.002)	**0.04**
HB **	0.983	(0.969–0.997)	**0.015**	0.994	(0.979–1.01)	0.469
LDH **	1.001	(1–1.001)	**0.008**	1	(1–1.001)	0.604
AP **	1	(1–1.001)	**0.07**	1.001	(1–1.001)	**0.032**

* Variables considered dichotomic; ** Variables considered continuous. ECOG: Eastern Cooperative Oncology Group; PSA: prostate-specific antigen; HB: hemoglobin concentration; LDH: lactate dehydrogenase; AP: alkaline phosphatase; HR: hazard ratio; CI: confidence interval. Significant *P*-Values are shown in bold.

**Table 5:** Contingency table showing the frequency of samples with *ARV7* and *ARFL* high and low expression according to treatments. N: number; AA: abiraterone; E: enzalutamide; OR: odds ratio, Fisher exact test; * *P* < 0.05.

	*ARV7*			*ARFL*		
Expression Levels	Low	High	Total	Low	High	Total
**Taxane cohort**	* *OR: 3.957; P = 0.011*			* *OR: 3.75, P = 0.007*		
**No AA/E pre-taxanes N(%)**	31 (57.4)	32 (84.2)	63 (68.5)	14 (48.3)	49 (77.8)	63 (68.5)
**AA/E pre-taxanes N(%)**	23 (42.6)	6 (15.8)	29 (31.5)	15 (51.7)	14 (22.2)	29 (31.5)
**Total**	54 (58.7)	38 (41.3)	92 (100)	29 (31.5)	63 (68.5)	92 (100)
**AA/E cohort**	***OR: 0.414*, *P = 0.199***			* *OR: 0.126, P = 0.040*		
**No taxanes pre-AA/E N(%)**	7 (50)	12 (29.3)	19 (34.5)	18 (41.9)	1 (8.3)	19 (34.5)
**Taxanes pre-AA/E N(%)**	7 (50)	29 (70.7)	36 (65.5)	25 (58.1)	11 (91.7)	36 (65.5)
**Total**	14 (24.4)	41 (74.5)	55 (100)	43 (78.2)	12 (21.8)	55 (100)
